# Does the length of stay in hospital affect healthcare outcomes of patients without COVID-19 who were admitted during the pandemic? A retrospective monocentric study

**DOI:** 10.1007/s11739-022-02945-7

**Published:** 2022-02-24

**Authors:** David Fluck, Christopher Henry Fry, Suzanne Rankin, Andrea Lewis, Jonathan Robin, Jacqui Rees, Jo Finch, Yvonne Jones, Gareth Jones, Julia Tudose, Liz Taylor, Thang Sieu Han

**Affiliations:** 1 Department of Cardiology, Ashford and St Peter’s Hospitals NHS Foundation Trust, Guildford Road, Chertsey, KT16 0PZ Surrey UK; 2 School of Physiology, Pharmacology and Neuroscience, University of Bristol, Bristol, BS8 1TD UK; 3 Department of Medicine, Ashford and St Peter’s Hospitals NHS Foundation Trust, Guildford Road, Chertsey, KT16 0PZ Surrey UK; 4 Department of Quality, Ashford and St Peter’s Hospitals NHS Foundation Trust, Guildford Road, Chertsey, KT16 0PZ Surrey UK; 5 Department of Endocrinology, Ashford and St Peter’s Hospitals NHS Foundation Trust, Guildford Road, Chertsey, KT16 0PZ Surrey UK; 6 Institute of Cardiovascular Research, Royal Holloway, University of London, Egham, TW20 0EX Surrey UK

**Keywords:** Older age, Mortality, Readmission, Length of stay, Coronavirus, Charlson comorbidity index

## Abstract

Uncertainties remain if changes to hospital care during the coronavirus disease (COVID-19) pandemic had an adverse impact on the care-quality of non-COVID-19 patients. We examined the association of hospital length of stay (LOS) with healthcare quality indicators in patients admitted with general medical conditions (non-COVID-19). In this retrospective monocentric study at a National Health Service hospital (Surrey), data were collected from 1st April 2019 to 31st March 2021, including the pandemic from 1st March 2020. Primary admissions, in-hospital mortality, post-discharge readmission and mortality were compared between the pre-pandemic (reference group) and pandemic period, according to LOS categories. There were 10,173 (47.7% men) from the pre-pandemic and 11,019 (47.5% men) from the pandemic period; mean (SD) age 68.3 year (20.0) and 68.3 year (19.6), respectively. During the pandemic, primary admission rates for acute cardiac conditions, pulmonary embolism, cerebrovascular accident and malignancy were higher, whilst admission rates for respiratory diseases and common age-related infections, and in-hospital mortality rates were lower. Amongst 19,721 survivors, sex distribution and underlying health status did not significantly differ between admissions before the pandemic and during wave-1 and wave-2 of the pandemic. Readmission rates did not differ between pre-pandemic and pandemic groups within the LOS categories of < 7 and 7–14 days, but were lower for the pandemic group who stayed > 14 days. For patients who died within seven days of admission, in-hospital mortality rates were lower in patients admitted during the pandemic. Mortality rates within 30 days of discharge did not differ between pre-pandemic and pandemic groups, irrespective of the initial hospital LOS. Despite higher rates of admission for serious conditions during the pandemic, in-hospital mortality was lower. Discharge time was similar to that for patients admitted before the pandemic, except it was earlier during the pandemic for those who stayed > 14 days, There were no group differences in quality-care outcomes.

**Supplementary Information:**

The online version contains supplementary material available at 10.1007/s11739-022-02945-7.

## Introduction

The coronavirus disease (COVID-19) pandemic had led to an abrupt transformation in healthcare systems [1, 2]. There was an urgent need for rapid modification and expansion of hospital ward structures [3, 4], mobilisation of healthcare professionals, and reorganisation of medical and surgical provisions [5, 6]. Consequently, there was a significant reduction in healthcare service delivery as well as service utilisation [7–11], which particularly affected older adults [12], patients with mental health disorders [13], and individuals of lower socioeconomic status [14, 15].

Since the beginning of the COVID-19 pandemic, there has been an unprecedented amount of research and publications devoted to the management of COVID-19 patients. However, comparable research on general medical conditions (non-COVID-19) has diminished, although the latter group comprises the majority (almost 90%) of all hospital admissions [5]. There has been a suggestion that many older people were generally discharged more rapidly during the pandemic [16]. Despite the huge challenges brought on by the pandemic, the National Health Service (NHS) has continued to maintain the highest possible standards of care for all patients admitted to hospital. However, uncertainties remain if changes adapted to hospital care for COVID-19 could have an adverse impact on quality of care for patients who presented with general medical conditions. We examined the association hospital length of stay (LOS), a measure of time to discharge, with healthcare quality indicators (post-discharge readmission and mortality) both in patients admitted with general medical conditions (non-COVID-19 related) during the COVID-19 pandemic in comparison to patients admitted during the immediately preceding year.

## Methods

### Study design, participants and setting

We analysed prospectively collected data of consecutive unplanned admissions to a single NHS hospital (Ashford and St Peter’s NHS Foundation Trust, Surrey, UK), comprising a reference group of patients admitted before the COVID-19 pandemic (1st April 2019 to 29th February 2020), and patients who presented with general medical conditions (without COVID-19) during the pandemic (1st March 2020 to 31st March 2021). Our centre is the largest provider of acute hospital services in Surrey County, serving a catchment area of 450,000 across the north-west of Surrey. There are 450 hospital beds with a broad range of medical specialties including the following: accident and emergency services, critical care, and emergency medical care which covers the majority of medical disciplines such as cardiology, respiratory medicine, gastroenterology, neurology, endocrinology, geriatrics, oncology and haematology (follow this link for complete list: https://www.ashfordstpeters.nhs.uk/specialties). This centre was appointed as hub for the care of COVID-19 patients.

### Selection criteria

From a total of 22,644 patients, there were 10,173 admitted before and 12,471 during the COVID-19 pandemic. Patients who were admitted with COVID-19 (*n* = 1452) were excluded, leaving 21,192 non-COVID-19 patients admitted before and during the pandemic period eligible for this study (Supplementary Fig. 1). The average rates of admission were 925 month before the pandemic and 959 months during the pandemic (848 month non-COVID-19 and 111 month COVID-19 patients).

### Measurements

Clinical data were recorded including age, sex and comorbidities (coded according to the international classification of diseases, ICD-11) [17]. Charlson comorbidity index (CCI) scores were calculated from comorbidities [18]. Information on care quality including the LOS, frequency of readmissions within 28 days and mortality in hospital and within 30 days of discharge from hospital was documented.

### Categorisation of variables

Age was grouped into bands of 18–39, 40–59, 60–79 and ≥ 80 years and CCI was categorised into three groups with scores of 0, 1 and ≥ 2. Frequencies of early readmissions were categorised either into a single readmission or ≥ 2 readmissions within 28 days of discharge. Hospital LOS was classified into the following three groups: < 7, 7–14 and > 14 days to indicate early, intermediate and late discharge from hospital.

### Statistical analysis

Chi-square and Fisher’s exact tests were used to examine differences between categorical variables including age bands, sex, CCI as well as cause of primary admission and outcomes including in-hospital mortality, readmission and mortality according to LOS categories. Logistic regression was used to assess the risk of readmission and mortality after hospital discharge (dependent variables) in patients admitted during the COVID-19 pandemic compared to those admitted before the pandemic (reference group). Data are presented as two models: model 1: unadjusted, and model 2: adjusted for age, sex, CCI and primary admissions. Odds ratios (OR) are given with 95% confidence intervals (CI). Analyses were performed using IBM SPSS Statistics, v25.0 (IBM Corp., Armonk, NY).

## Results

### Characteristics of pre-pandemic and pandemic groups

A total of 21,192 non-COVID-19 patients were studied as follows: 10,173 (47.7% men) from the pre-pandemic and 11,019 (47.5% men) from the pandemic period; mean (SD) age 68.3 years (20.0) and 68.3 years (19.6), respectively. Compared to pre-pandemic group, the LOS in hospital during the pandemic was shorter by 1.3 days (95% confidence interval: 1.0–1.6, *P* < 0.001), and primary admissions during the pandemic were proportionally higher for acute coronary syndrome and acute myocardial infarct, pulmonary embolism, cerebrovascular accident, and malignancy. By contrast, admission rates for respiratory conditions and common infections such as chronic obstructive pulmonary disease, asthma and pneumonia, pneumonia, urinary tract infection and sepsis were significantly lower. There were no group differences in admissions for congestive heart failure, brain trauma, gastrointestinal conditions including inflammatory bowel disease and gastrointestinal haemorrhage, or diabetes mellitus (Table 1).

**Table 1:** Characteristics of 21,192 patients admitted before and of those admitted during the COVID-19 pandemic

	Proportion of patients (%)		Group differences (*P* values)
	Before pandemic (*n* = 10,173)	During pandemic (*n* = 11,019)	
Age on admission (year)			
18–39	11.8	10.8	0.008
40–59	19.6	20.8	
60–79	32.9	33.7	
≥ 80	35.8	34.8	
Sex			
Men	47.7	47.5	0.358
Women	52.3	52.5	
Primary admissions			
Acute coronary syndrome	0.6	0.8	0.018
Acute myocardial infarct	3.7	4.2	0.021
Pulmonary embolism	1.1	1.4	0.022
Cerebrovascular accident	4.6	5.2	0.023
Malignancy	2.3	2.8	0.015
Chronic obstructive pulmonary disease	2.1	1.7	0.019
Asthma	1.2	0.6	< 0.001
Pneumonia	10.0	5.1	< 0.001
Urinary tract infection	4.9	4.3	0.015
Sepsis	1.8	1.3	0.001
Congestive heart failure	1.8	2.1	0.070
Brain trauma	1.0	0.8	0.161
Inflammatory bowel disease	0.8	0.7	0.398
Gastrointestinal haemorrhage	1.1	1.0	0.372
Diabetes mellitus	1.5	1.4	0. 358
Charlson comorbidity index scores			
0	86.3	86.0	0.077
1	10.2	9.9	
≥ 2	3.5	4.1	
Length of stay in hospital			
< 7 days	73.3	75.3	< 0.001
7–14 days	11.7	13.6	
> 14 days	15.0	111.1	
Mortality in hospital	7.6	6.0	< 0.001
Mortality within 30 days of discharge	4.6	4.1	0.025
Readmissions within 28 days of discharge			
No readmission	88.1	89.9	< 0.001
Single readmission	9.1	7.7	
≥ 2 readmissions	2.8	2.4	

There were 1434 in-hospital deaths, and 19,721 patients who survived to discharge, 9401 (47.6% men) from pre-pandemic and 10,320 (47.3% men) from COVID-19 pandemic period, with mean (SD) age of 67.2 years (20.2) and 67.4 years (19.7), respectively. Compared to patients admitted before the pandemic, those admitted during the pandemic had similar sex distribution or CCI scores, but proportionally more for those aged 40–79 years, whilst the rates of readmission within 28 days (11.9% vs 10.1%, *P* < 0.001) and mortality within 30 days of hospital discharge (4.6% vs 4.1%, *P* = 0.025) were lower. There were higher proportions of patients with LOS < 7 days (75.7% versus 77.3%), 7–14 days (10.9% vs 12.8%), and conversely there was a lower proportion with LOS > 14 days (13.3% vs 9.9%) (Table 1).

### Outcomes differences between pre-pandemic and pandemic groups according to LOS in hospital

**Fig. 1.**
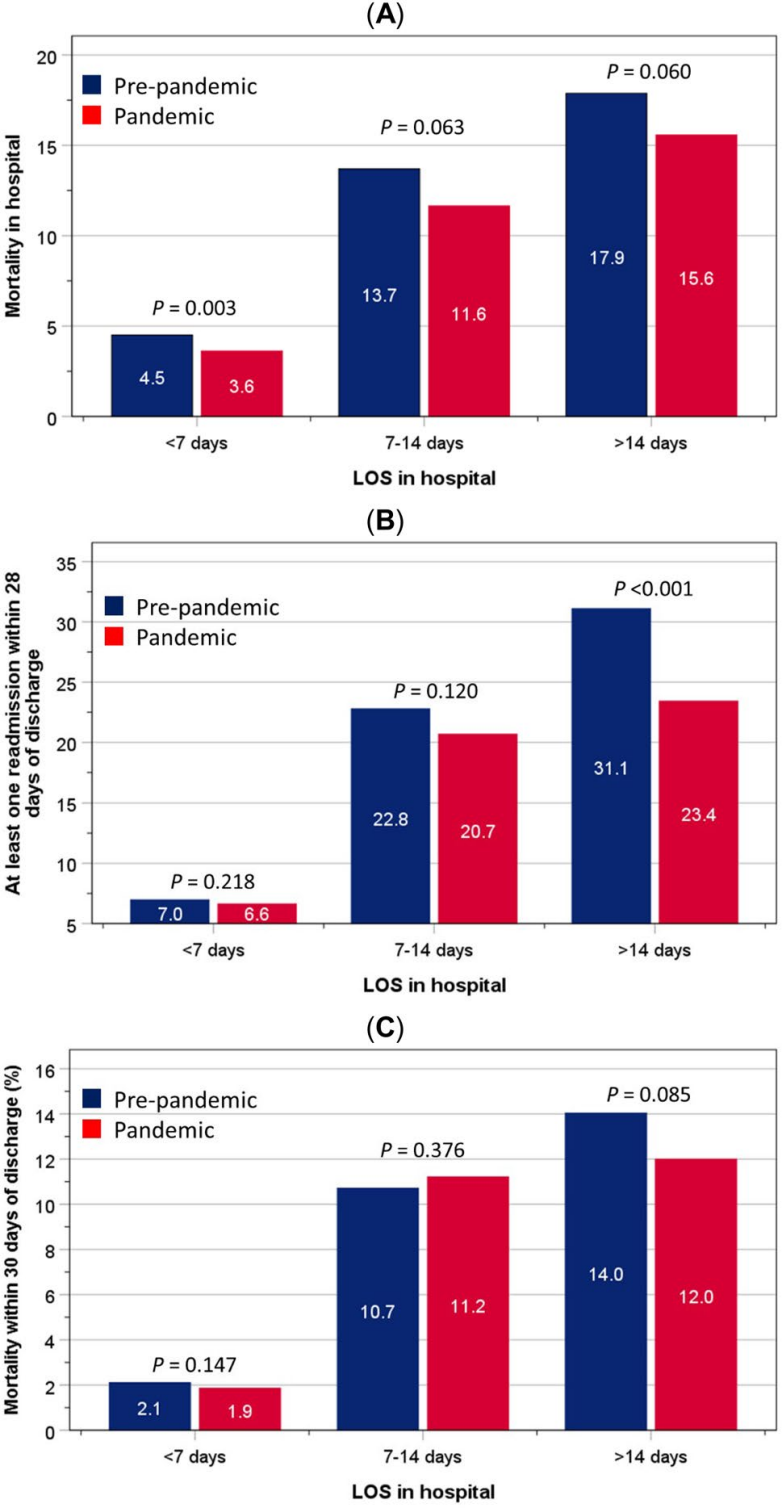
In-hospital mortality rates (**A**), readmission within 28 days (**B**), and mortality within 30 days (**C**) of hospital discharge, according to LOS amongst patients admitted before the pandemic and those admitted during the pandemic

In-hospital mortality rate during the pandemic (6.0%) was significantly (*χ*^2^ = 20.4, *P* < 0.001) lower than that before the pandemic (7.6%). In-hospital mortality rates rose incrementally with longer LOS in hospital in both study groups (pre-pandemic: *χ*^2^ = 394.9, *P* < 0.001; during pandemic: *χ*^2^ = 363.5, *P* < 0.001). Generally, in-hospital mortality rates were lower for any given LOS in hospital (Fig. 1A). The age of patients who died in hospital before the pandemic (mean = 81.9 years, SD = 11.8) did not differ (*P* = 0.186) from that of patients who died during the pandemic (mean = 81.1 years, SD = 12.4).

Amongst patients with LOS < 7 days in hospital, the proportions of early readmission within the pre-pandemic group were 7%, rising to 22.8 and 31.1% amongst those who stayed in hospital 7–14, and > 14 days, respectively (*χ*^2^ = 721.6, *P* < 0.001). The corresponding figures for those admitted during the pandemic group were 6.6, 20.7 and 23.4% (*χ*^2^ = 468.6, *P* < 0.001). There were no group differences in the rates of readmission within LOS categories of < 7 days and 7–14 days. By contrast, with the LOS category of > 14 days, and compared to the reference group, there was a lower proportion of patients admitted during the pandemic who were readmitted at least once within 28 days of discharge (31.1% vs 23.4%, *P* < 0.001) (Fig. 1B).

Similarly, the rates of mortality within 30 days of discharge were lowest (2.1%) amongst those admitted before the pandemic (reference) and with the shortest LOS (< 7 days). This increased to 10.7 and 14% for those who stayed in hospital 7–14, and > 14 days, respectively (*χ*^2^ = 439.2, *P* < 0.001). The corresponding figures for those who were admitted during the pandemic were 1.9, 11.2 and 12.0% (*χ*^2^ = 438.7, *P* < 0.001). There were no significant differences in the rates of mortality within 30 days of hospital discharge between pre-pandemic and pandemic groups who stayed in hospital < 7 days (2.1% vs 1.9%, *P* = 0.147), 7–14 days (10.7% vs 11.2%, *P* = 0.376), or > 14 days (14.0% vs 12.0%, *P* = 0.085) (Fig. 1C).

**Fig. 2.**
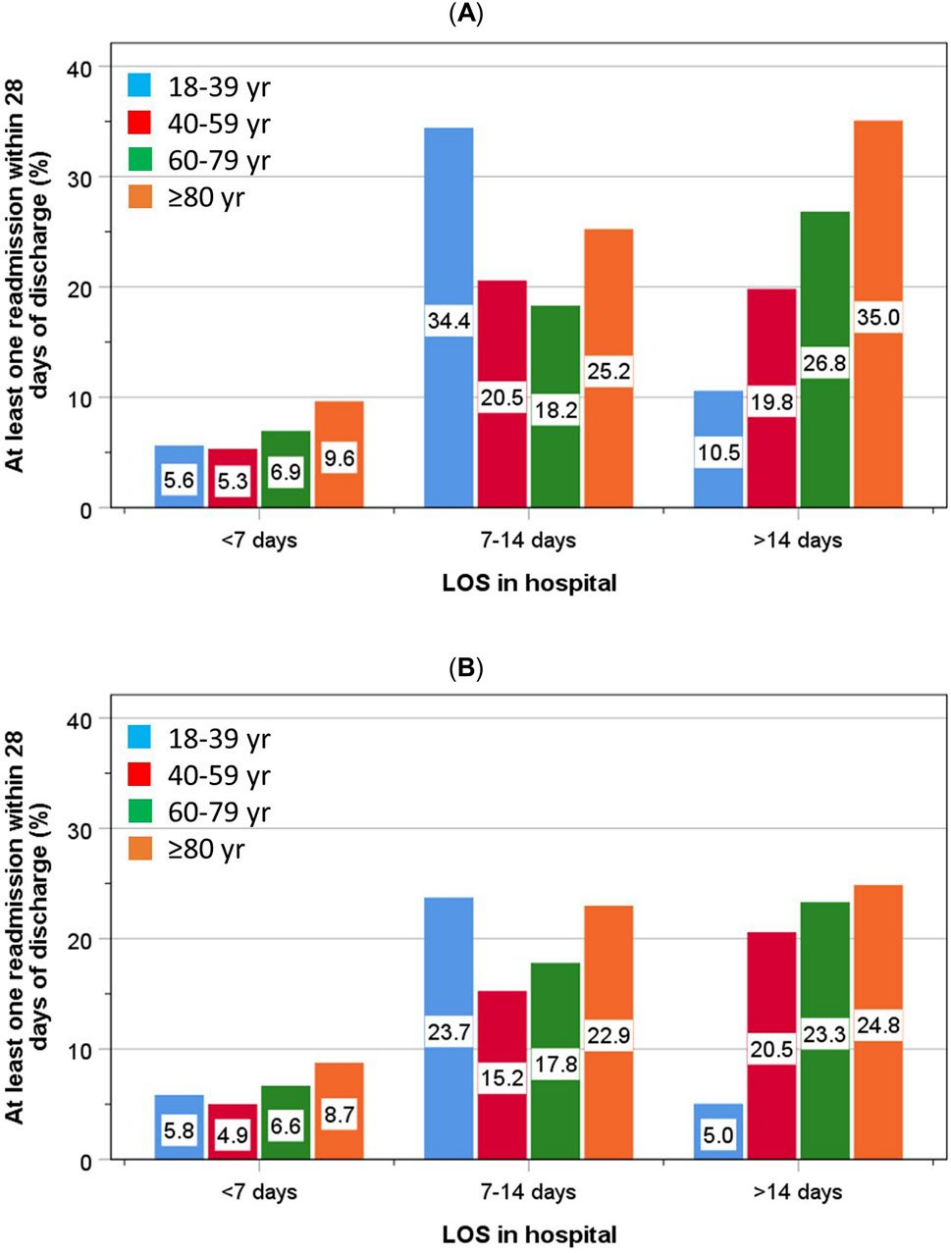
Distribution of readmissions at least once within 28 days of discharge amongst patients admitted before (**A**) and during the pandemic (**B**) according to age

**Fig. 3.**
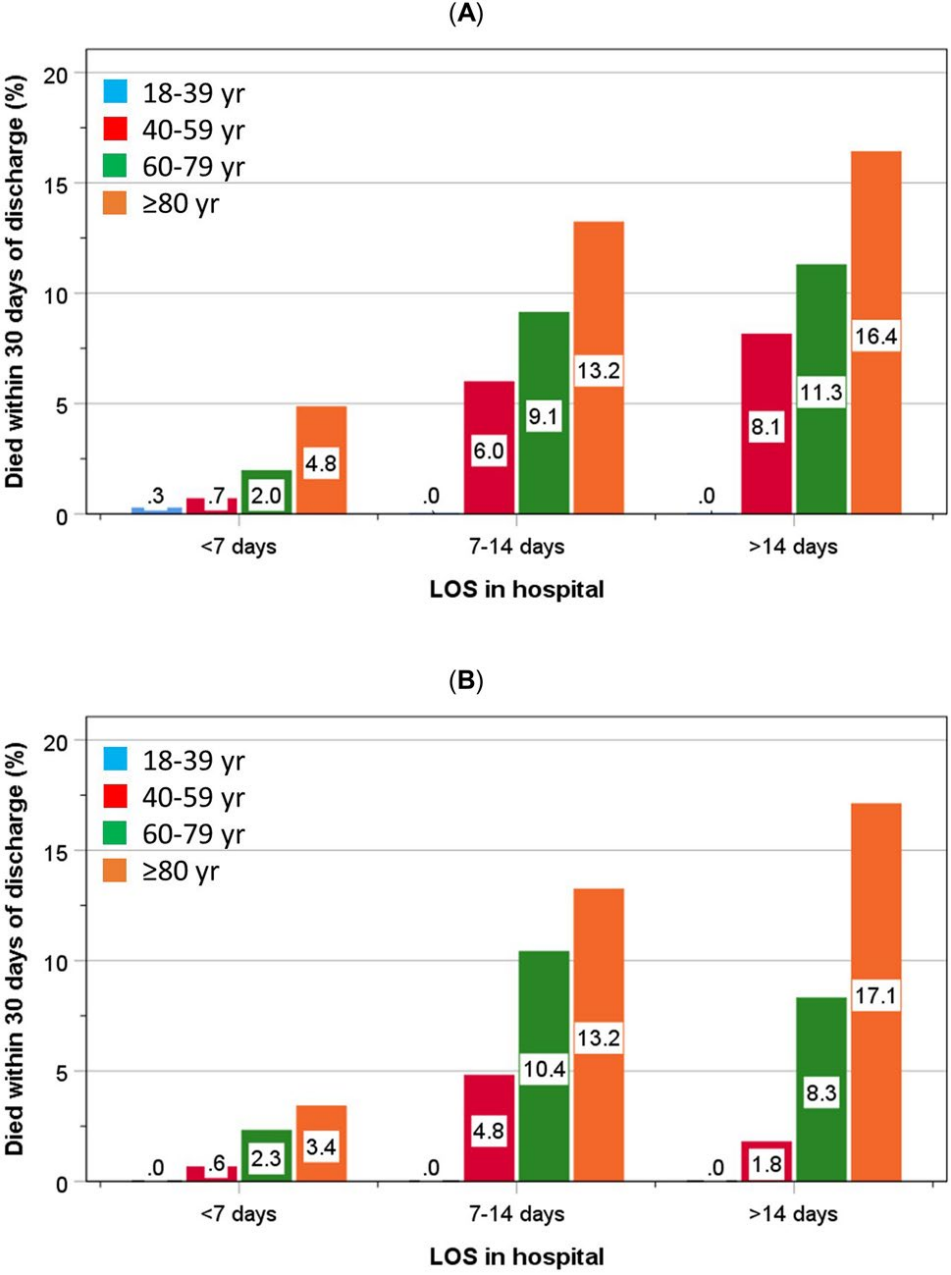
Distribution of mortality within 30 days of discharge amongst patients admitted before (**A**) and during the pandemic (**B**) according to age

Within each category of LOS, there was an increasing trend with greater age for readmission within 28 days of discharge (Fig. 2A, B) and of mortality within 30 days of discharge (Fig. 3A, B). This was duplicated by both the pre-pandemic and the pandemic groups, except for a surge in readmissions amongst the youngest group within the 7–14 days LOS category. In general, the rates of readmission and mortality were lower amongst patients who were admitted during the pandemic within each age category (Figs. 2, 3).

**Fig. 4.**
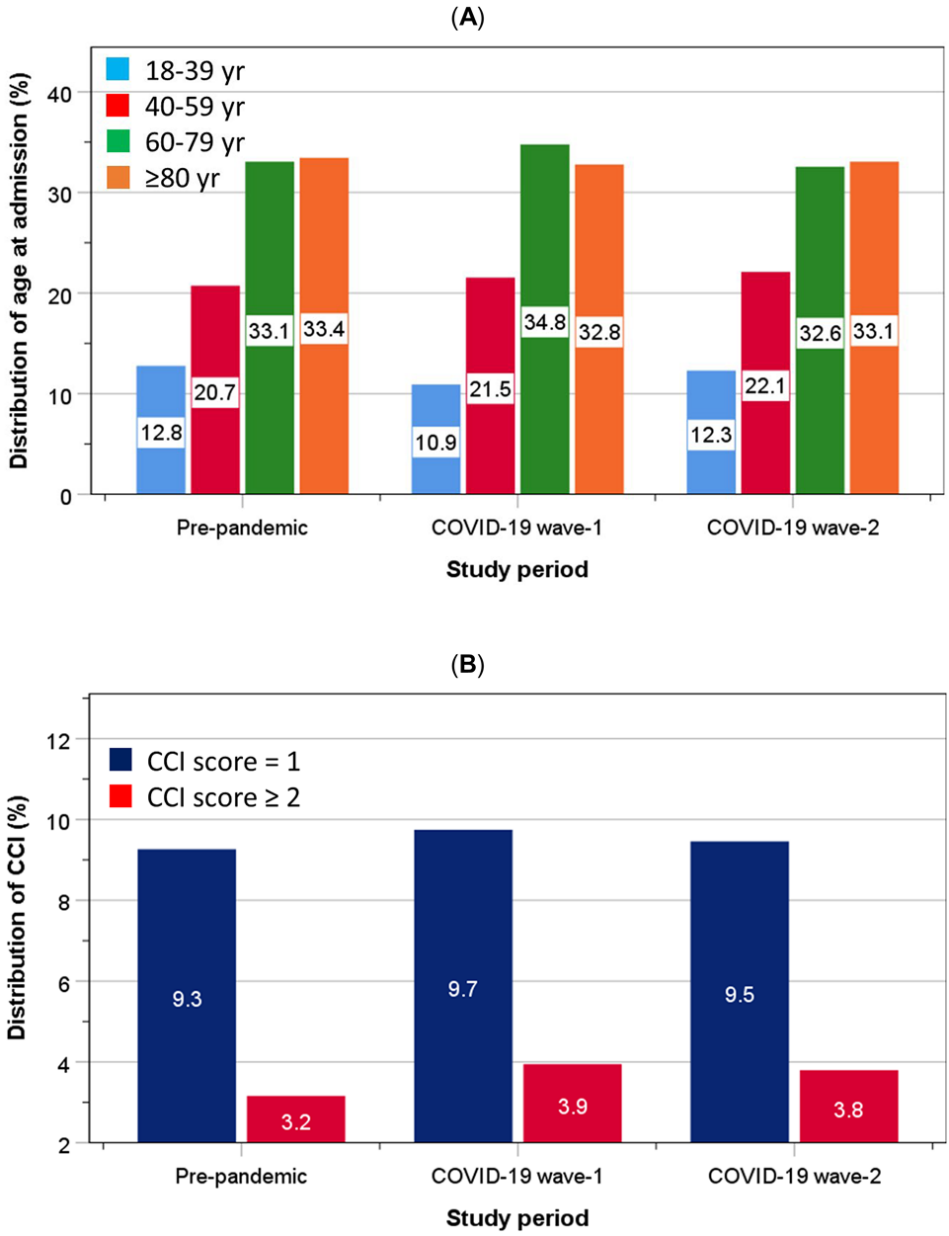
Distribution of age (**A**) and Charlson comorbidity index (**B**) at admission before, during wave-1 and wave-2 COVID-19 pandemic

The distributions of age (Fig. 4A) and CCI (Fig. 4B) did not differ substantially between patients admitted before the pandemic and those admitted during either wave-1 or wave-2 of the pandemic.

Logistic regression analysis with adjustment for age, sex, CCI and primary admissions showed that compared with patients who were admitted before the COVID-19 pandemic, patients who were admitted during the pandemic had similar risk of readmission and mortality after a discharge from hospital, irrespective of the LOS. The only exception was of a lower risk of in-hospital mortality for those who died in hospital within 7 days of admission and for readmission within 28 days of discharge for those who stayed in hospital > 14 days (Table 2).

**Table 2:** Logistic regression to assess the risk of in-hospital mortality, post-discharge readmission and mortality amongst patients admitted during the pandemic compare to those admitted before the pandemic (reference group)

In-hospital mortality	Risk from admission during COVID-19 pandemic compared to admission before the pandemic (reference group)					
	Unadjusted			Adjusted for age, sex, CCI, and primary admissions^a^		
	OR	95%CI	*P*	OR	95%CI	*P*
LOS < 7 days	0.79	0.68–0.93	0.005	0.80	0.67–0.95	0.010
LOS = 7–14 days	0.83	0.66–1.04	0.112	0.88	0.69–1.12	0.288
LOS > 14 days	0.85	0.69–1.04	0.109	0.92	0.75–1.14	0.462
Readmitted within 28 days of discharge	OR	95%CI	*P*	OR	95%CI	*P*
LOS < 7 days	0.95	0.84–1.08	0.417	0.95	0.84–1.08	0.466
LOS = 7–14 days	0.88	0.73–1.08	0.220	0.91	0.74–1.11	0.329
LOS > 14 days	0.68	0.56–0.82	< 0.001	0.71	0.58–0.86	< 0.001
Mortality within 30 days of discharge						
LOS < 7 days	0.88	0.70–1.11	0.269	0.87	0.69–1.11	0.271
LOS = 7–14 days	1.05	0.81–1.37	0.700	1.05	0.80–1.38	0.718
LOS > 14 days	0.84	0.65–1.07	0.153	0.91	0.71–1.10	0.489

*CCI*, Charlson comorbidity index

^a^primary admissions including 15 conditions presented in Table 1

## Discussion

### Summary

Compared to pre-pandemic period, for patients with non-COVID related conditions during the pandemic period, the admission rates for serious conditions such as acute cardiac conditions, pulmonary embolism, cerebrovascular accident and malignancy were higher, whilst those for respiratory diseases and common age-related infections and in-hospital mortality were lower. There was no evidence for a risk of early readmission or short-term mortality amongst those who were discharged early (hospital LOS < 7 days): this was irrespective of age, sex and CCI. Our findings, therefore, indicate that standards of care were maintained for non-COVID-19 patients admitted to our hospital during the pandemic, and continued to fulfil the standards set by the Getting It Right First Time programme. As far as we are aware, no such research findings have been reported in current literature.

### LOS and care-quality outcomes

The findings of a higher proportion of patients with early discharge (LOS < 7 days) and a lower proportion with late discharge (LOS > 14 days) during the pandemic, whilst care-quality outcomes (readmission and mortality) were not compromised, suggest that efficiency of discharge planning may have improved, as well as greater efforts were made in the management of patients. In addition, we followed the national guidelines with the implementation of surge rota [20], redeployment of staff [21] and deferral of annual leave [22]. Similar to other centres, there was a restructure and expansion of hospital wards such as critical care units [23], and a shift from face-to-face outpatient clinics to telephone clinics [5]. These measures were likely to have a positive impact on overall ability to maintain non-COVID cover. There was also a reduction of non-emergency surgical activity, but we did not need to divert any patients to other hospitals.

In line with national guidelines, there were a number of initiatives carried out at our hospital during the pandemic. These included improvement in hospital discharge and community support which was made possible by a national discharge fund provided by the government from 19th March 2020 until 31st March 2022. This fund covered partially the cost of recovery and support services, rehabilitation and reablement care after hospital discharge, with an intention to assist more people to return to their home [19]. The improved community care after the first COVID-19 pandemic played a pivotal role in reducing hospital readmissions. This includes implementation of the Enhanced Health in Care Homes service to support staff and residents during the pandemic [24]. Admissions and readmissions for many ambulatory care-sensitive and urgent care-sensitive conditions such as chronic obstructive pulmonary disease, asthma and diabetes could be avoided with timely and effective community care. A recent report has indicated that emergency admissions were reduced for ambulatory care-sensitive conditions by 24% and urgent care-sensitive conditions by 17% during the pandemic compared to the previous year [25]. The observation in our study of lower rates of admission for respiratory diseases (chronic obstructive pulmonary disease and asthma) and common infections associated with older age (pneumonia, urinary tract infection and sepsis), and no change in congestive heart failure and diabetes, during the pandemic is consistent with the findings in the report above. On the other hand, admission rates were higher during the pandemic for serious conditions which cannot be managed in the community including cardiac conditions (acute coronary syndromes and acute myocardial infarction), pulmonary embolism, cerebrovascular accidents and malignancies. The changes in cause of admission during the pandemic were accompanied by a reduction of in-hospital mortality, indicating the overall quality of care was maintained for non-COVID patients, despite higher proportions of patients who were admitted with serious conditions.

Furthermore, there was an increase in infection control globally, including hygiene and personal protective equipment, that resulted in a very large decrease of nosocomial infections, such as *Clostridium difficile* infections and improved antibiotic treatments [26, 27]. Consequently, there was a reduction of LOS in the most vulnerable groups of patients such as oncology patients [27].

A number of other changes have also been observed that could have an impact on admission and readmission to hospital in many parts of the world. There was a very large reduction [28, 29], or even abolition [30] of influenza admissions. This is likely to result from better hygiene and social distancing and behavioural changes of individuals from fear of contracting COVID-19 infection in hospital [31]. Furthermore, in the UK and other affluent countries [32], family support may have been more readily available since there were more people on furlough schemes [33], which may have helped to allow older patients to be discharged from hospital earlier.

### Patient characteristics

The similarity in distributions of age, sex and underlying health status (CCI) in patients admitted before the pandemic and those admitted during the pandemic indicates that self-selection for hospital admission was not apparent. This suggests that if patients were acutely unwell, irrespective of their age and underlying health status, they were equally likely to be admitted to hospital during the pandemic as those before the pandemic. These findings were confirmed by multivariable logistic regression with adjustments for age, sex and CCI, showing no evidence for an increase in readmission or mortality amongst patients admitted during the pandemic compared to those admitted before the pandemic (reference group), within early, intermediate or late discharge group. These findings were corroborated by evidence that the numbers of patients admitted with general medical conditions was reduced with the surge of COVID-19 admissions with each pandemic wave [5]. However, there was no evidence for a decrease in the proportions of older adults or those with poorer health status (CCI) during either wave-1 or wave-2 of the COVID-19 pandemic. Overall, the present study found that early discharge before or during the pandemic was associated with better outcomes, which are consistent with previous findings [34].

There were two distinct waves of the COVID-19 pandemic. Similar to national data, COVID-19 admissions to our hospital surged rapidly in wave-1. By contrast, COVID-19 admissions in wave-2 were more gradual, but the total number of admissions were much higher. We have demonstrated that non-COVID admissions mirrored closely the peaks and troughs of COVID-19 admissions [5].

In addition to higher proportions of patients admitted with serious conditions during the pandemic, in-hospital mortality rates were lower overall with any given hospital LOS (higher burden of comorbidities and frailty), whilst the age of those who died was virtually identical to those admitted before the pandemic. Therefore, the relationship between LOS and post-discharge outcomes were probably not affected by conditions from primary admission or in-hospital mortality, i.e. bias from healthier or survivors is unlikely.

### Strengths and limitations

The strengths of this study lies in its large numbers of patients who were consecutively admitted to the same centre, and the use of a control group of patients who admitted in the year immediately before the pandemic. The study covered a wide range of age and underlying health status assessed by standardised indices. There is, however, a lack of long-term outcome data, which are not yet available. Caution should be taken if extrapolating our findings to other centres as management of patients during the COVID-19 pandemic may differ between these centres, particularly in other countries where health services are not centrally funded by the government.

In conclusion, despite the higher rates of admission for serious conditions during the pandemic, in-hospital mortality was lower, with discharge time being similar to that of patients admitted before the pandemic, except earlier for those who stayed > 14 days during the pandemic, whilst there were no group differences in quality care outcomes.

## Supplementary Information

Below is the link to the electronic supplementary material.

Supplementary file1 (DOCX 113 KB)

## Abbreviations

CCI: Charlson comorbidity index
CI: Confidence interval
COVID-19: Coronavirus disease-19
ICD: International classification of diseases
LOS: Length of stay
NHS: National Health Service
OR: Odds ratio
SD: Standard deviation
