# Supplementary figures and images for: Does the length of stay in hospital affect healthcare outcomes of patients without COVID-19 who were admitted during the pandemic? A retrospective monocentric study
Source: Intern Emerg Med. 2022 Feb 24;17(5):1385–93. doi: 10.1007/s11739-022-02945-7 (PMC8869351; doi:10.1007/s11739-022-02945-7)

**Supplentary Figure 1.** Flow chart showing selection criteria for analysis in the present study.

**
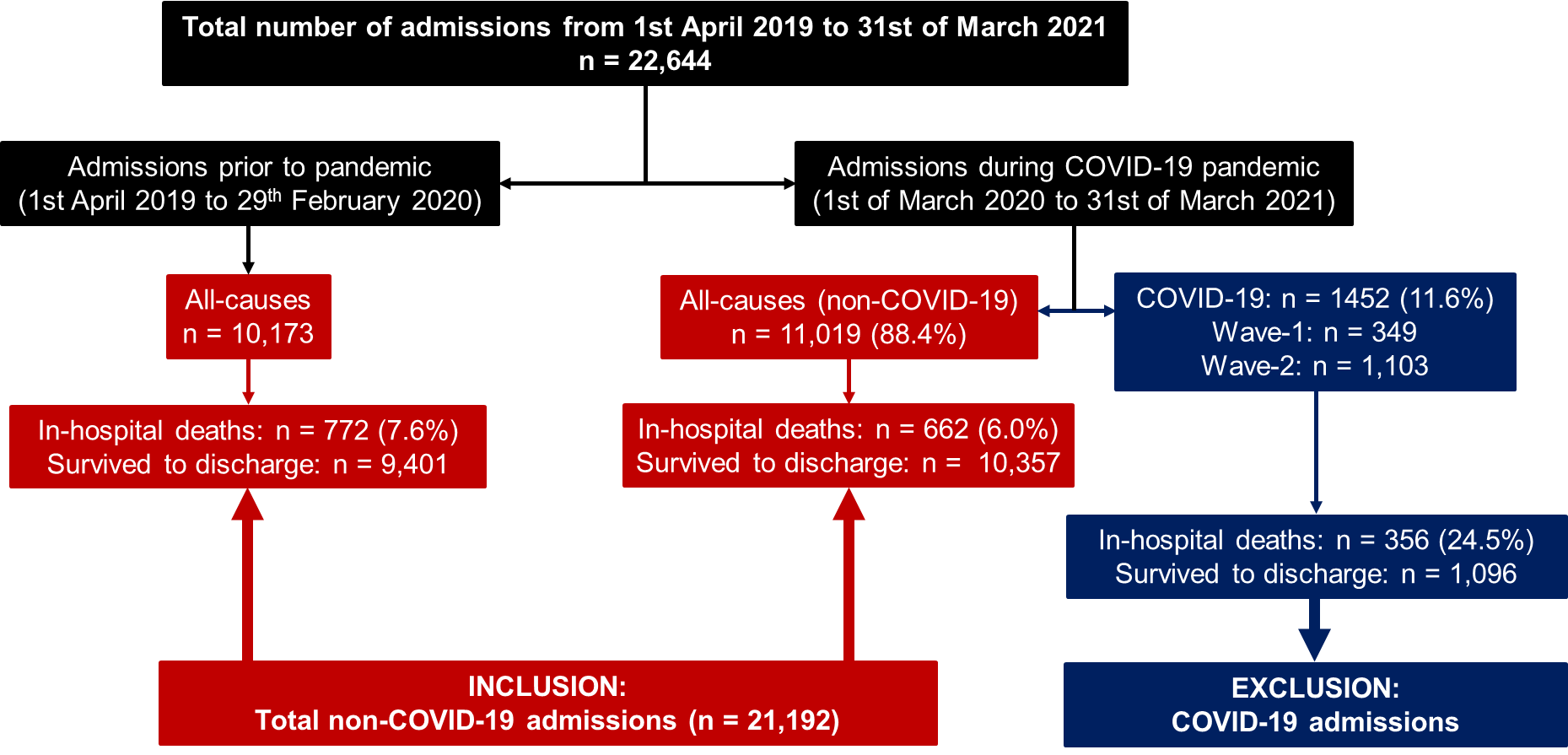
**

Supplement: Supplementary file 1 — Supplementary file1 (DOCX 113 KB) [file 11739_2022_2945_MOESM1_ESM.docx]
